# Supplementary material for: Modeling glioblastoma heterogeneity as a dynamic network of cell states
Source: Mol Syst Biol. 2021 Sep 16;17(9):e10105. doi: 10.15252/msb.202010105 (PMC8444284; doi:10.15252/msb.202010105)
Supplement: Supplementary file 6 — Source Data for Figure 5 [file MSB-17-e10105-s004.zip › Figure5A_sourcedata/GSEA_3071/hallmarks_stateIV.GseaPreranked.1623742551702/HALLMARK_MITOTIC_SPINDLE.html]

Details for gene set HALLMARK\_MITOTIC\_SPINDLE[GSEA]

|  || Dataset | state53071 |
| Phenotype | NoPhenotypeAvailable |
| Upregulated in class | na\_neg |
| GeneSet | HALLMARK\_MITOTIC\_SPINDLE |
| Enrichment Score (ES) | -0.3826886 |
| Normalized Enrichment Score (NES) | -2.0226967 |
| Nominal p-value | 0.0 |
| FDR q-value | 0.0015507612 |
| FWER p-Value | 0.014 |
Table: GSEA Results Summary

  

Fig 1: Enrichment plot: HALLMARK\_MITOTIC\_SPINDLE      
 Profile of the Running ES Score & Positions of GeneSet Members on the Rank Ordered List

  

| PROBE | GENE SYMBOL | GENE\_TITLE | RANK IN GENE LIST | RANK METRIC SCORE | RUNNING ES | CORE ENRICHMENT || 1 | FGD4 |  |  | 65 | 1.758 | 0.0110 | No |
| 2 | CCDC88A |  |  | 135 | 1.429 | 0.0168 | No |
| 3 | SPTBN1 |  |  | 387 | 0.918 | -0.0193 | No |
| 4 | PREX1 |  |  | 429 | 0.873 | -0.0156 | No |
| 5 | APC |  |  | 492 | 0.822 | -0.0165 | No |
| 6 | DLG1 |  |  | 520 | 0.798 | -0.0111 | No |
| 7 | MID1 |  |  | 708 | 0.655 | -0.0384 | No |
| 8 | MYO9B |  |  | 822 | 0.592 | -0.0522 | No |
| 9 | SHROOM2 |  |  | 828 | 0.589 | -0.0453 | No |
| 10 | NIN |  |  | 869 | 0.574 | -0.0454 | No |
| 11 | KIF3C |  |  | 885 | 0.567 | -0.0407 | No |
| 12 | PPP4R2 |  |  | 905 | 0.558 | -0.0369 | No |
| 13 | FARP1 |  |  | 931 | 0.549 | -0.0344 | No |
| 14 | RABGAP1 |  |  | 998 | 0.523 | -0.0401 | No |
| 15 | FSCN1 |  |  | 1003 | 0.521 | -0.0339 | No |
| 16 | NCK1 |  |  | 1070 | 0.502 | -0.0399 | No |
| 17 | CLIP2 |  |  | 1105 | 0.490 | -0.0399 | No |
| 18 | KIF1B |  |  | 1143 | 0.482 | -0.0406 | No |
| 19 | ARHGEF12 |  |  | 1172 | 0.473 | -0.0397 | No |
| 20 | KLC1 |  |  | 1196 | 0.466 | -0.0379 | No |
| 21 | ARL8A |  |  | 1219 | 0.458 | -0.0360 | No |
| 22 | DOCK4 |  |  | 1247 | 0.452 | -0.0352 | No |
| 23 | ARHGEF3 |  |  | 1295 | 0.441 | -0.0383 | No |
| 24 | KIFAP3 |  |  | 1301 | 0.440 | -0.0334 | No |
| 25 | NUMA1 |  |  | 1338 | 0.431 | -0.0346 | No |
| 26 | ARFGEF1 |  |  | 1353 | 0.428 | -0.0316 | No |
| 27 | PCM1 |  |  | 1458 | 0.400 | -0.0463 | No |
| 28 | CYTH2 |  |  | 1494 | 0.390 | -0.0478 | No |
| 29 | ARHGEF7 |  |  | 1536 | 0.381 | -0.0506 | No |
| 30 | MARCKS |  |  | 1713 | 0.348 | -0.0799 | No |
| 31 | PLEKHG2 |  |  | 1723 | 0.346 | -0.0770 | No |
| 32 | HOOK3 |  |  | 1751 | 0.339 | -0.0777 | No |
| 33 | ABR |  |  | 1947 | 0.306 | -0.1112 | No |
| 34 | KIF3B |  |  | 2255 | 0.261 | -0.1669 | No |
| 35 | RASA1 |  |  | 2437 | -0.262 | -0.1983 | No |
| 36 | KIF5B |  |  | 2471 | -0.266 | -0.2011 | No |
| 37 | LMNB1 |  |  | 2487 | -0.268 | -0.2004 | No |
| 38 | TUBGCP3 |  |  | 2952 | -0.326 | -0.2856 | No |
| 39 | SMC1A |  |  | 2968 | -0.328 | -0.2841 | No |
| 40 | SMC4 |  |  | 3035 | -0.337 | -0.2923 | No |
| 41 | SASS6 |  |  | 3091 | -0.344 | -0.2983 | No |
| 42 | CDC42EP2 |  |  | 3294 | -0.374 | -0.3322 | No |
| 43 | AKAP13 |  |  | 3299 | -0.375 | -0.3280 | No |
| 44 | SHROOM1 |  |  | 3324 | -0.379 | -0.3276 | No |
| 45 | PCNT |  |  | 3374 | -0.387 | -0.3318 | No |
| 46 | FGD6 |  |  | 3432 | -0.396 | -0.3375 | No |
| 47 | NET1 |  |  | 3471 | -0.404 | -0.3395 | No |
| 48 | PIF1 |  |  | 3519 | -0.413 | -0.3430 | No |
| 49 | CDC42EP4 |  |  | 3556 | -0.420 | -0.3443 | No |
| 50 | ARHGDIA |  |  | 3578 | -0.423 | -0.3427 | No |
| 51 | CEP57 |  |  | 3694 | -0.443 | -0.3590 | No |
| 52 | WASF2 |  |  | 3818 | -0.469 | -0.3764 | Yes |
| 53 | KIF22 |  |  | 3832 | -0.472 | -0.3726 | Yes |
| 54 | NOTCH2 |  |  | 3853 | -0.477 | -0.3701 | Yes |
| 55 | ECT2 |  |  | 3902 | -0.487 | -0.3728 | Yes |
| 56 | CDC27 |  |  | 3918 | -0.491 | -0.3691 | Yes |
| 57 | SMC3 |  |  | 3952 | -0.498 | -0.3688 | Yes |
| 58 | INCENP |  |  | 3985 | -0.507 | -0.3682 | Yes |
| 59 | CENPF |  |  | 4003 | -0.511 | -0.3647 | Yes |
| 60 | CDC42 |  |  | 4045 | -0.524 | -0.3656 | Yes |
| 61 | CDC42EP1 |  |  | 4048 | -0.525 | -0.3589 | Yes |
| 62 | NEK2 |  |  | 4086 | -0.536 | -0.3589 | Yes |
| 63 | CCNB2 |  |  | 4103 | -0.542 | -0.3547 | Yes |
| 64 | CENPJ |  |  | 4148 | -0.555 | -0.3558 | Yes |
| 65 | KNTC1 |  |  | 4176 | -0.561 | -0.3535 | Yes |
| 66 | PLK1 |  |  | 4205 | -0.569 | -0.3513 | Yes |
| 67 | BUB1 |  |  | 4246 | -0.578 | -0.3513 | Yes |
| 68 | KIF15 |  |  | 4324 | -0.603 | -0.3581 | Yes |
| 69 | ARF6 |  |  | 4425 | -0.643 | -0.3687 | Yes |
| 70 | EPB41 |  |  | 4449 | -0.654 | -0.3644 | Yes |
| 71 | SAC3D1 |  |  | 4462 | -0.660 | -0.3579 | Yes |
| 72 | ARHGAP10 |  |  | 4486 | -0.672 | -0.3534 | Yes |
| 73 | FLNB |  |  | 4496 | -0.677 | -0.3460 | Yes |
| 74 | PDLIM5 |  |  | 4612 | -0.739 | -0.3583 | Yes |
| 75 | BIRC5 |  |  | 4618 | -0.745 | -0.3493 | Yes |
| 76 | RACGAP1 |  |  | 4623 | -0.748 | -0.3401 | Yes |
| 77 | TTK |  |  | 4644 | -0.762 | -0.3337 | Yes |
| 78 | MID1IP1 |  |  | 4653 | -0.765 | -0.3251 | Yes |
| 79 | NUSAP1 |  |  | 4654 | -0.765 | -0.3148 | Yes |
| 80 | EZR |  |  | 4714 | -0.803 | -0.3155 | Yes |
| 81 | KIF4A |  |  | 4727 | -0.812 | -0.3069 | Yes |
| 82 | KIF20B |  |  | 4745 | -0.825 | -0.2992 | Yes |
| 83 | CENPE |  |  | 4757 | -0.832 | -0.2901 | Yes |
| 84 | KIF11 |  |  | 4760 | -0.835 | -0.2794 | Yes |
| 85 | KIF23 |  |  | 4825 | -0.891 | -0.2798 | Yes |
| 86 | PRC1 |  |  | 4846 | -0.912 | -0.2714 | Yes |
| 87 | PALLD |  |  | 4851 | -0.914 | -0.2600 | Yes |
| 88 | DLGAP5 |  |  | 4857 | -0.922 | -0.2486 | Yes |
| 89 | STK38L |  |  | 4898 | -0.963 | -0.2435 | Yes |
| 90 | AURKA |  |  | 4906 | -0.973 | -0.2318 | Yes |
| 91 | FBXO5 |  |  | 4910 | -0.978 | -0.2193 | Yes |
| 92 | EPB41L2 |  |  | 4974 | -1.051 | -0.2174 | Yes |
| 93 | FLNA |  |  | 4975 | -1.051 | -0.2033 | Yes |
| 94 | TUBA4A |  |  | 5010 | -1.111 | -0.1950 | Yes |
| 95 | KIF2C |  |  | 5028 | -1.150 | -0.1829 | Yes |
| 96 | TOP2A |  |  | 5029 | -1.152 | -0.1675 | Yes |
| 97 | TPX2 |  |  | 5049 | -1.184 | -0.1553 | Yes |
| 98 | NDC80 |  |  | 5055 | -1.200 | -0.1402 | Yes |
| 99 | BRCA2 |  |  | 5069 | -1.222 | -0.1264 | Yes |
| 100 | SORBS2 |  |  | 5124 | -1.342 | -0.1188 | Yes |
| 101 | MYH9 |  |  | 5132 | -1.366 | -0.1019 | Yes |
| 102 | CDK1 |  |  | 5164 | -1.512 | -0.0877 | Yes |
| 103 | ANLN |  |  | 5183 | -1.577 | -0.0701 | Yes |
| 104 | VCL |  |  | 5190 | -1.611 | -0.0497 | Yes |
| 105 | BIN1 |  |  | 5267 | -2.432 | -0.0318 | Yes |
| 106 | ARHGAP29 |  |  | 5279 | -2.693 | 0.0021 | Yes |
Table: GSEA details [plain text format]

  

Fig 2: HALLMARK\_MITOTIC\_SPINDLE: Random ES distribution      
 Gene set null distribution of ES for **HALLMARK\_MITOTIC\_SPINDLE**
